# Supplementary material for: Determining zebrafish dorsal organizer size by a negative feedback loop between canonical/non-canonical Wnts and Tlr4/NFκB
Source: Nat Commun. 2023 Nov 8;14:7194. doi: 10.1038/s41467-023-42963-3 (PMC10632484; doi:10.1038/s41467-023-42963-3)
Supplement: Supplementary file 1 — Supplementary Information [file 41467_2023_42963_MOESM1_ESM.pdf]

Determining zebrafish dorsal organizer size by a negative feedback loop between canonical/non-canonical Wnts and Tlr4/NFκB

Zou et al.

Supplementary information:
Supplementary Figure 1-7 and table 1-2

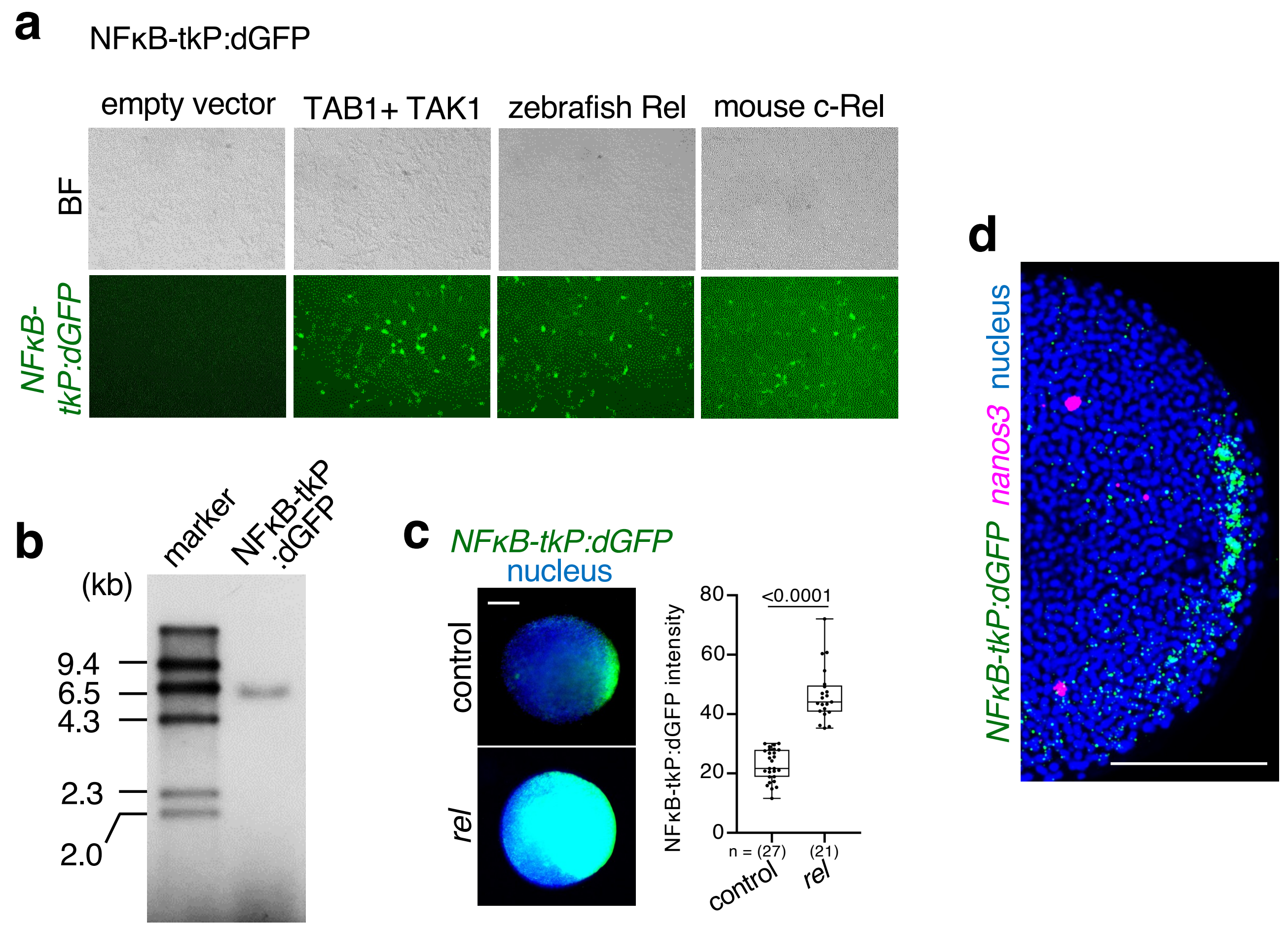

Supplementary Fig. 1: Generation of NFκB-tkP:dGFP reporter zebrafish.

a Activation of the NFκB signalling stimulates the activities of NFκB-tkP:dGFP reporter. HEK293 cells were transfected transiently with empty vector and expression vectors encoding zebrafish Rel, mouse c-Rel, and the NFκB signalling activators TAB1 and TAK1[100] as indicated. Bright-field (BF) images are shown in the top panels. dGFP-expressing cells (bottom panels, green) were visualized by fluorescence microscopy. **b** Southern blot analysis of the transgene in NFκB-tkP:dGFP transgenic zebrafish line. Genomic DNA was prepared from the tail fins of adult fishes and used for Southern blot analysis. **c** *rel* overexpression leads to activation of NFκB reporter in zebrafish. Fluorescent *in situ* hybridization (FISH) for *dGFP* (green) in NFκB-tkP:dGFP-transgenic embryos injected with control (mKO2) or *rel* mRNA at dome stage. Nucleus (blue) is stained by Hoechst33342. Animal views with dorsal to the right. Scale bar = 200 µm. Box plots of dGFP intensity show first and third quartile, median is represented by a line, whiskers indicate the minimum and maximum. Each dot represents one embryo. P-values for unpaired two-tailed t-tests are indicated. **d** NFκB-activated cells are not germ cells. Double fluorescent *in situ* hybridization (FISH) for *dGFP* (green) and *nanos3* (germ cell marker, megenta) in NFκB-tkP:dGFP-transgenic embryos at dome stage. Animal views with dorsal to the right. Scale bar = 200 µm. Nucleus (blue) is stained by Hoechst33342. Source data are provided as a Source Data file.

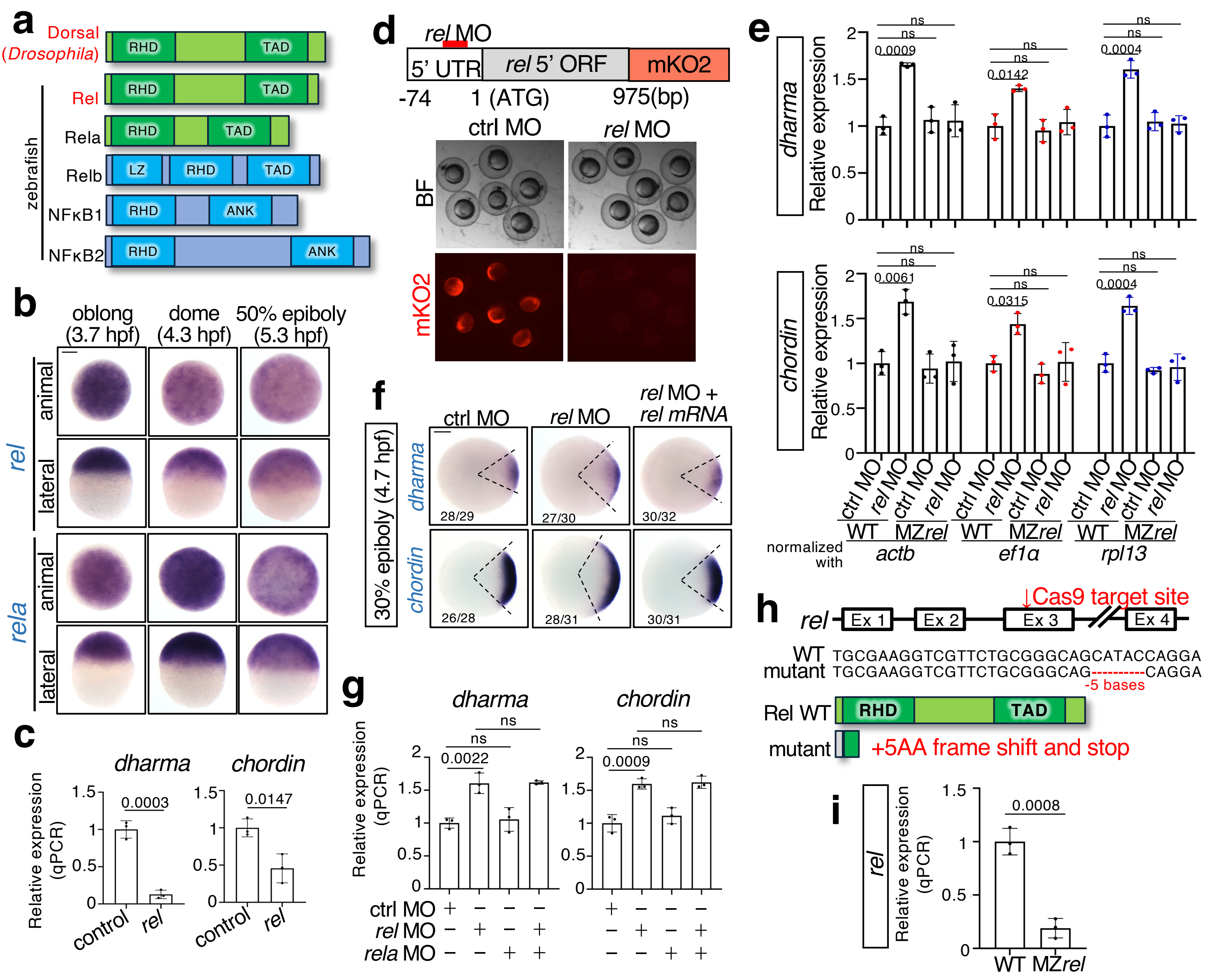

Supplementary Fig. 2: MO-mediated knockdown and CRISPR/Cas9-mediated knockout of a zebrafish NFκB homologue gene *rel*.

a Schematic diagrams of zebrafish NFκB homologues and *Drosophila* Dorsal protein. RHD: Rel homology domain, ANK: ankyrin repeat, TAD: transactivation domain, LZ: leucine zipper. **b** *rel* and *rela* mRNA are ubiquitously expressed in early embryos. WISH for *rel* and *rela* in zebrafish embryos at indicated stages, animal view. Scale bar = 200 µm. **c** *rel* overexpression significantly reduces dorsal marker gene expression. qPCR analysis for expression of *dharma* and *chordin* of embryos injected with control (mKO2) or *rel* mRNA at 30% epiboly. Normalized values are shown as means ± SEM. n = 3, biologically independent samples. Unpaired two-tailed t-test. **d** *rel* MO blocks translation of *rel* gene. To examine the effect of *rel* MO, mRNA including the 5'UTR and 5'coding region (-74–975 bp) of the *rel* gene fused in-frame with mKO2 gene (5'UTR-*rel*-hmKO2) was injected into zebrafish embryos with *rel* MO. The *rel* MO annealing site is indicated by the red line. Representative images (lower panel) show that injection of *rel* MO, but not ctrl MO, strongly diminished the translation of 5'UTR-*rel*-hmKO2 mRNA. Cells expressing mKO2 (red) were visualized by fluorescence microscopy. Bright-field (BF) images are shown in top panels. **e** *rel* MO significantly enhanced dorsal marker gene expression in wild-type (WT) but not MZ*rel* embryos. qPCR analysis for *dharma*, *chordin* in WT and MZ*rel* embryos injected with ctrl MO, *rel* MO at 30% epiboly. Normalized values with different standard genes are shown as mean ± SEM. n = 3 biologically independent samples. P-values for two-tailed one-way ANOVAs with Sidak correction are indicated. **f** *rel* overexpression rescues *rel* MO-induced dorsal expansion. WISH for *dharma* and *chordin* in embryos injected with ctrl MO, *rel* MO or *rel* MO with *rel* mRNA at 30% epiboly stage, animal view. **g** Rel but not Rela is the main NFκB which restricts dorsal organizer formation. qPCR analysis for *dharma*, *chordin* in embryos injected with ctrl MO, *rel* MO and *rela* MO at 30% epiboly. Normalized values with different standard genes are shown as mean ± SEM. n = 3 biologically independent samples. P-values for two-tailed one-way ANOVAs with Sidak correction are indicated. **h, i** Generation of *rel* knockout by CRISPR/Cas9. (h) Genomic organization of *rel* locus and Cas9-targeted regions are shown in top panels. An indel mutation in the third exon of *rel* gene following a deletion of 5 nucleotides was generated. Frameshift and premature termination of translation in the *rel* mutant transcript are shown in the bottom panel. (i) *rel* mRNA levels were significantly decreased in MZ*rel* embryos. qPCR analysis for expression of *rel* in MZ*rel* or WT embryos at sphere stage. Normalized values are shown as means ± SEM. n = 3, biologically independent samples. P-value for an unpaired two-tailed t-test is indicated. Source data are provided as a Source Data file.

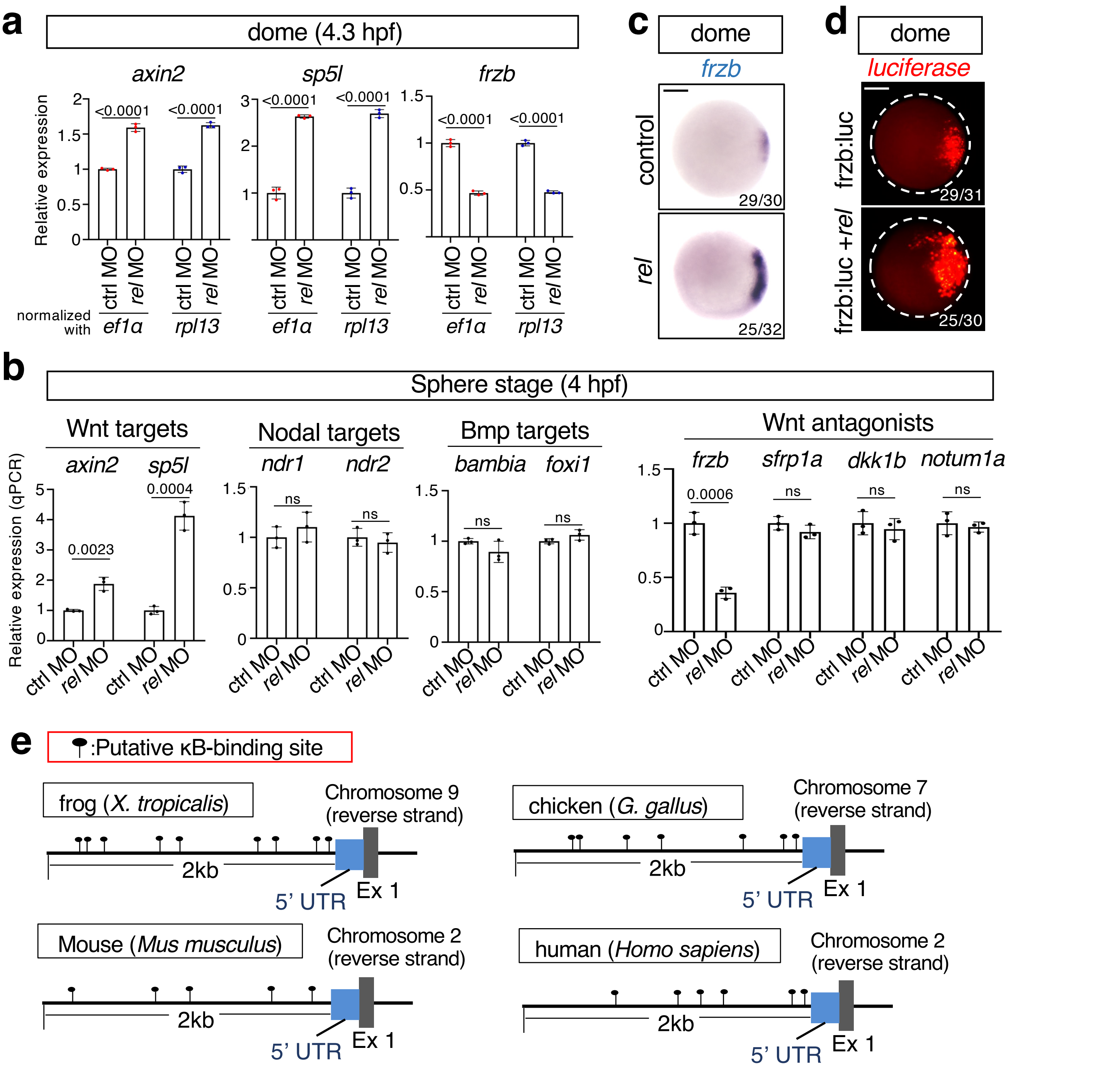

Supplementary Fig. 3: Rel positively regulates the Wnt antagonist Frzb which might be evolutionarily conserved. **a** Rel negatively regulates Wnt/β-catenin signalling. qPCR analysis for Wnt targets (*axin2* and *sp5l*) and *frzb* in embryos injected with ctrl MO or *rel* MO at dome stage (4.3 hpf). Normalized values with different standard genes are shown as mean ± SEM. n = 3 biologically independent samples. P-values for unpaired two-tailed t-tests are indicated. **b** Rel negatively regulates Wnt/β-catenin signalling whereas doesn't affect Nodal and BMP signalling at 4 hpf. qPCR analysis for Wnt targets (*axin2* and *sp5l*), Nodal targets (*ndr1* and *ndr2*), BMP targets (*foxi1* and *bambia*) and Wnt antagonists (*frzb*, *sfrp1a*, *dkk1b* and *notum1a*) in embryos injected with ctrl MO or *rel* MO at sphere stage (4 hpf). Normalized values are shown as means ± SEM. n = 3, biologically independent samples. P-values for unpaired two-tailed t-tests are indicated. **c, d** *rel* overexpression enhanced *frzb* expression. (c) WISH for *frzb* in dome stage embryos injected with control (mKO2) mRNA or *rel* mRNA, animal view. (d) FISH for *luciferase* (red) in dome-stage embryos injected with frzb:luc, with *rel* mRNA as indicated, animal view. Scale bar = 200 μm. **e** Putative NFκB-binding elements in the *frzb* promoter of other vertebrates. Schematic diagrams of the upstream regions of frog (*Xenopus tropicalis*), chicken (*Gallus gallus*), mouse (*Mus musculus*) and human (*Homo sapiens*) *frzb* genes. We analysed 2 kb upstream of 5'UTR regions using the NFκB-binding elements search tool (http://thebrain.bwh.harvard.edu/nfkb/) and discovered several elements which show the high z-score and thus possess strong potential to bind to Rel homodimer. The elements are marked with black "pins". Grey and blue boxes indicate Exons and UTRs, respectively. Source data are provided as a Source Data file.

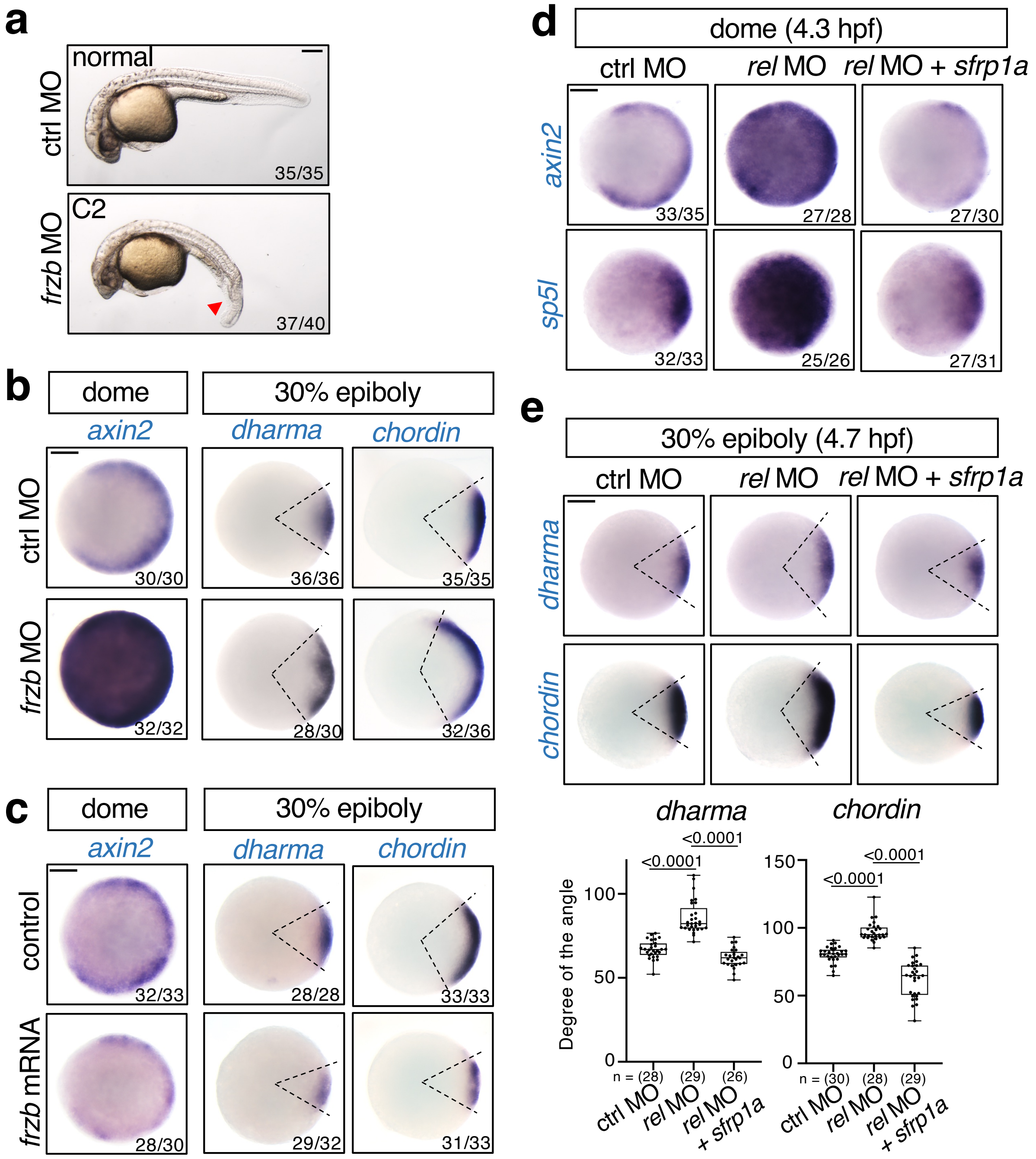

Supplementary Fig. 4: Negative regulation of Wnt/β-catenin signalling restricts dorsal organizer formation.
a Phenotypes of 27 hpf larvae injected with ctrl MO and *frzb* MO. Lateral views with anterior to the left. The loss of ventral tail fin is indicated with red arrowheads. The strength of dorsalization was scored. **b, c** WISH for *axin2*, *dharma* and *chordin* in embryos injected with (b) ctrl MO and *frzb* MO or (c) control (mKO2) or *frzb* mRNA at indicated stages. Animal views. Scale bar = 200 µm. **d, e** Overexpression of *sfrp1a* restored *rel* MO-induced phenotypes. WISH for (d) Wnt targets *axin2* and *sp5l*; (e) *dharma* and *chordin* in embryos injected with ctrl MO, *rel* MO or *rel* MO with *sfrp1a* mRNA at indicated stage, animal view. Box plots of the angle of marker genes show first and third quartile, median is represented by a line, whiskers indicate the minimum and maximum. Each dot represents one embryo. P-values for two-tailed one-way ANOVAs with Sidak correction are indicated. Source Data are provided as a Source Data file.

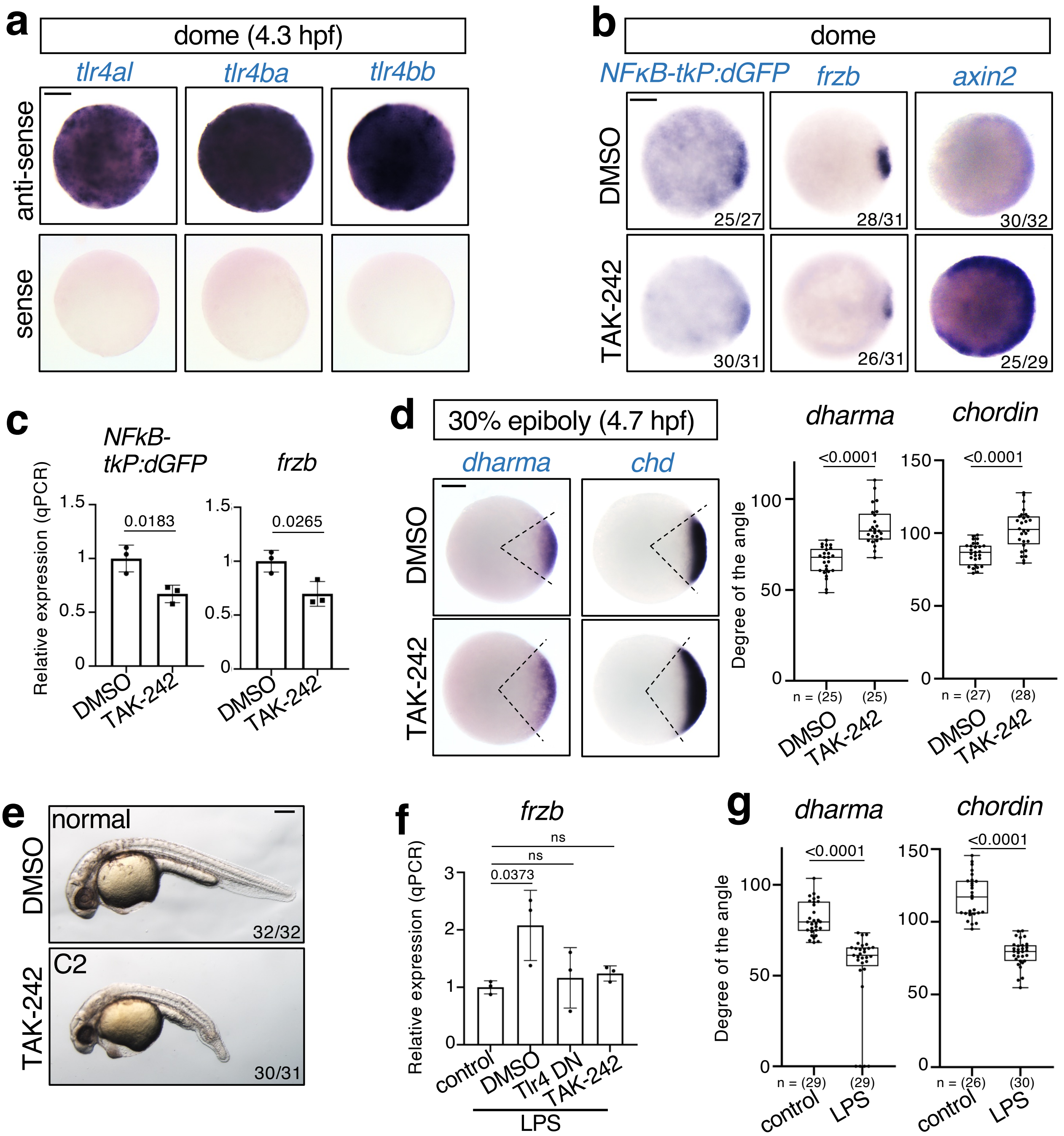

Supplementary Fig. 5: Tlr4/NFκB negatively regulates Wnt/β-catenin signalling and restricts dorsal organizer formation.

a *tlr4* is ubiquitously expressed in early embryos. WISH for *tlr4al*, *tlr4ba*, and *tlr4bb* with both anti-sense and sense probes at the dome stage. **b–e** Pharmacological inhibition of Tlr4 reduces *frzb* expression and enhances Wnt/β-catenin signalling and dorsal organizer formation. Embryos were treated with DMSO or TAK-242 from 3 hpf to (b, c) dome stage or (d, e) 30% epiboly stage. (b) WISH and (c) qPCR analysis for *dGFP* in NFκB-tkP:dGFP-transgenic embryos; *frzb*, *axin2* in WT embryos at dome stage. In b, animal views are shown. In c, normalized values are shown as means ± SEM. n = 3, biologically independent samples. P-values for unpaired two-tailed t-tests are indicated. (d) WISH for *dharma* and *chordin* in WT embryos at the indicated stage. Animal views. Box plots of the angle of marker genes show first and third quartile, median is represented by a line, whiskers indicate the minimum and maximum. Each dot represents one embryo. P-values for unpaired two-tailed t-tests are indicated. (e) Representative images of 27 hpf larvae. The strength of dorsalization was scored. Lateral views with anterior to the left. Scale bar = 200 µm. **f, g** Forced activation of Tlr4 by injection of lipopolysaccharide (LPS) activates NFκB signalling and inhibits dorsal marker gene expression. (f) qPCR analysis for *frzb* in embryos injected with LPS and treated with DMSO or TAK-242 or co-injected with Tlr4 DN, uninjected as control. DMSO and TAK-242 were treated from 3 hpf to dome stage. Normalized values with different standard genes are shown as mean ± SEM. n = 3 biologically independent samples. P-values for two-tailed one-way ANOVAs with Sidak correction are indicated. (g) Quantification of the expression of *dharma* and *chordin* in Fig. 5f. Box plots of the angle of marker genes in show first and third quartile, median is represented by a line, whiskers indicate the minimum and maximum. Each dot represents one embryo. P-values for unpaired two-tailed t-tests are indicated. Source data are provided as a Source Data file.

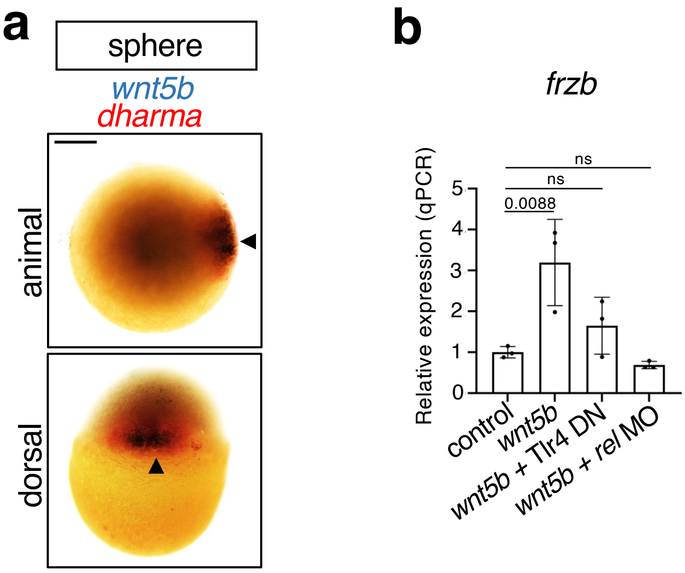

Supplementary Fig. 6: Wnt5b activates NFκB through Tlr4.

a *wnt5b* is specifically expressed in the dorsal region of early embryos. Double *in situ* hybridization for *wnt5b* (dark purple) and *dharma* (red) at sphere stage, animal view and dorsal view. The *wnt5b*-expressing dorsal regions are indicated with black arrowheads. Scale bar = 200 µm. **b** Wnt5b activates *frzb* expression through Tlr4/Rel. qPCR analysis for *frzb* in embryos injected with control (mKO2) or *wnt5b* mRNA, and co-injected with Tlr4 DN mRNA or *rel* MO. Normalized values with different standard genes are shown as mean ± SEM. n = 3 biologically independent samples. P-value for a two-tailed one-way ANOVA with Sidak correction is indicated. Source data are provided as a Source Data file.

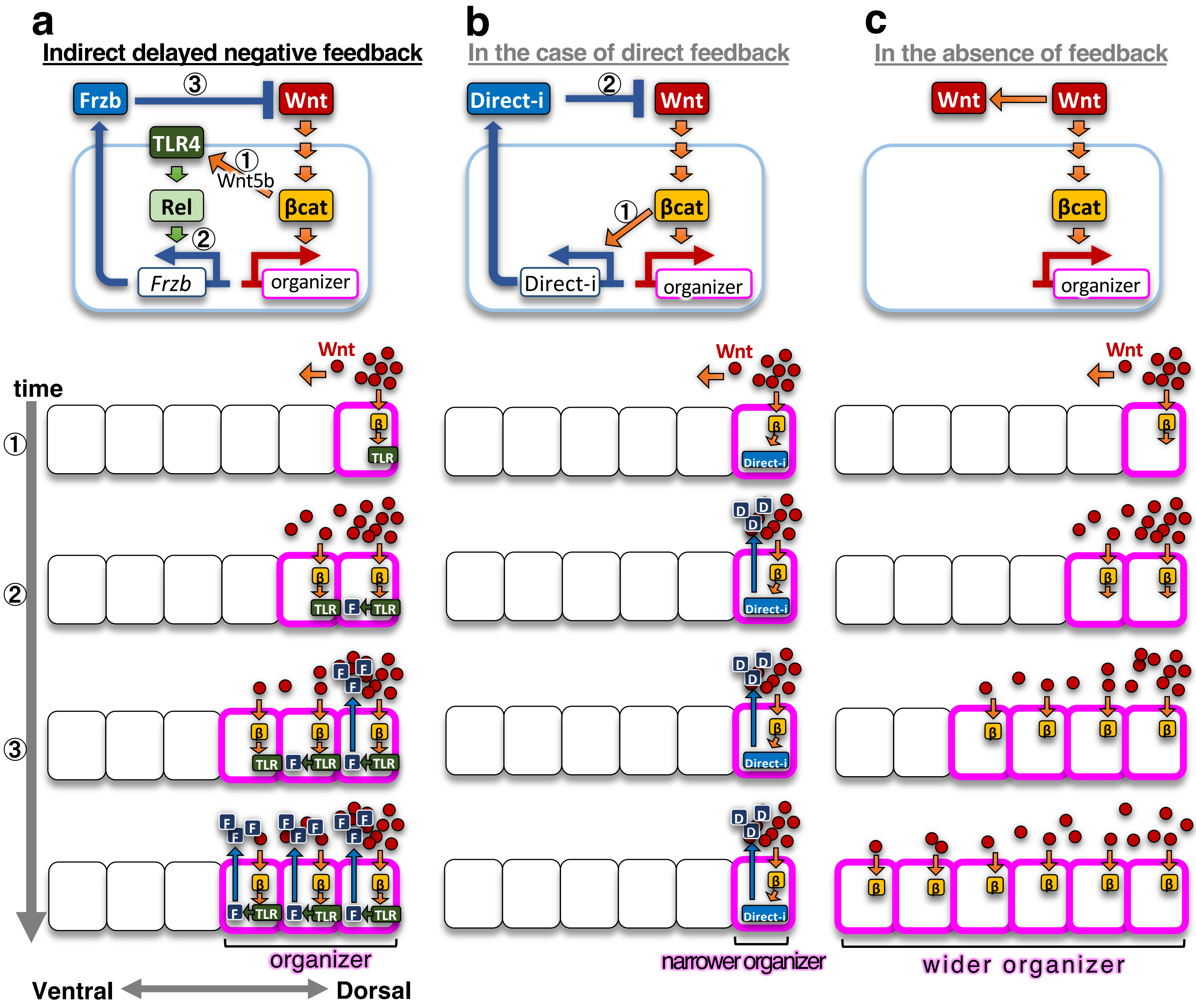

Supplementary Fig. 7: Models of the significance of the indirect delayed negative feedback in the formation of correct size of organizer.
a The indirect negative feedback would delay the timing of Wnt inhibition, which may ensure adequate duration for Wnt diffusion and consequent formation of organizer of proper size. **b** If Wnt signalling is inhibited through direct negative feedback during organizer induction, Wnt signalling would be immediately shut off and unable to form organizer of adequate size. **c** In the absence of negative feedback, Wnt would be diffused over a greater range and then produce an abnormally wide organizer.

Supplementary Table 1

MO sequences (5' to 3')

rel	TTGAGAGGGATTGCACATCCATAAC
rela	CCCACTGGTGAAACATTCCGTCCAT
frzb	GAGTTGATAGAAGAATGACATGCG
wnt5b	GTCCTTGGTTCATTCTCACATCCAT
ctnnb2	CCTTTAGCCTGAGCGACTTCCAAAC
standard control	CCTCTTACCTCAGTTACAATTTATA

Primers for qPCR (5' to 3')

dharma (Fw)	ACACCAGCAGGCAAACAGCA
dharma (Rv)	CAACCGGCTACGGCATAAGG
chordin (Fw)	ACGCCTGCTGCCATACAAT
chordin (Rv)	CACTGAGGGTCCACCGAGA
rel (Fw)	ACTACAGCTCCCAACAGCCTCAAA
rel (Rv)	AAACTGGTAGCCCGTTGCTAGTGA
rela (Fw)	CATTCCCTACGGCTAAACGA
rela (Rv)	AGAAAAAGGAGGTGGGTGGA
axin2 (Fw)	CTTAAACCTGCCACTAAGACCT
axin2 (Rv)	CATTCTCCTCCATAGCCGTC
sp5l (Fw)	ATTTCTTACAGGACCGCAC
sp5l (Rv)	CACGGTGAAGGTCATCTGGT
frzb (Fw)	AAAATAGGCCGGAAAGTAAAGC
frzb (Rv)	AGTGGTGGTTCCATTTGGTC
sfrp1a (Fw)	GATACTTCCACCTCCGAACCC
sfrp1a (Rv)	GATCCATGTTCTCCCGCTTG
dkk1b (Fw)	AATGACCCTGACATGATTCAGC
dkk1b (Rv)	AGGCTTGCAGATTTTGGACC
notum1a (Fw)	CACCTGTAACGACGGGACTC
notum1a (Rv)	GCATTCCTGTGCCTGTCTTG
ndr1 (Fw)	CTCCGTCTTGAGCCTCGTCG
ndr1 (Rv)	TCGCTGGACGTCATCGCTTG
ndr2 (Fw)	AATGCATACCGGTGCGAGGG
ndr2 (Rv)	GCAGGAACACGACTGGGGTG
bambia (Fw)	AGAGGGGACTCAACAGACCG
bambia (Rv)	CATTCGCAACGCCAGCATAA
foxi1 (Fw)	AAGTTGCACGGGATGAGGATGA
foxi1 (Rv)	CAGACTGGAAGTGTCCGCCAAT
d2EGFP (Fw)	AGGAGCGCACCATCTTCTT
d2EGFP (Rv)	GATGTTGTGGCGGATCTTG
actb1 (Fw)	TGGACTTTGAGCAGGAGATGGGAA
actb1 (Rv)	AAGGTGGTCTCATGGATACCGCAA
ef1a (Fw)	AGAAGGAAGCCGCTGAGATGG
ef1a (Rv)	TCCGTTCTTGGAGATACCAGCC
rpl13 (Fw)	TAAGGACGGAGTGAACAACCA
rpl13 (Rv)	CTTACGTCTGCGGATCTTTCTG

Supplementary Table 2

Primers for RNA probes

d2EGFP (Fw)	AAAGGATCCATCATGGTGAGCAAGGGCGAG	Vector: pCS2p+
d2EGFP (Rv)	AAATCTAGATTACTTGTACAGCTCGTCCATGCCG	
wnt5a (Fw)	TCTTTTTGCAGGATCGGATCCATGATGCTGCTGAAGCTGAAGTGG	
wnt5a (Rv)	CGAATCGATGGGATCTCACTTGCAGACGTACTGGTCC	
wnt5b (Fw)	TCTTTTTGCAGGATCGGATCCATGGATGTGAGAATGAACCAAGG	
wnt5b (Rv)	CGAATCGATGGGATCCTACTTGCACACAAACTGGTCTACG	
axin2 (Fw)	AAGTCGCACAGTTTGGAACC	Vector: pCRII-TOPO
axin2 (Rv)	CACATCATCGGCTATTGGCT	
sp5l (Fw)	GTTTCCCAGCCACATGCAAC	
sp5l (Rv)	ATGCTCCCATCGCAACCATT	
nanos3 (Fw)	ATGGGTTTGGCGGACATGAT	
nanos3 (Rv)	TGTTTTTGAGTGCGGTTGCG	
rel (Fw)	ATGGATGTGCAATCCCTCTC	
rel (Rv)	CTTGTGTCGTGGTTTCTGATG	
tlr4al (Fw)	ACAGATCACCTGGACAGCAAG	
tlr4al (Rv)	CGACAGTCTTTTCGCAGGGT	
tlr4ba (Fw)	GCCATTACTTTCAAGATTTCCGT	
tlr4ba (Rv)	GCAGCTCTAAAAGCCTTGCAT	
tlr4bb (Fw)	CTTTGACATTGACTACTGTGTGC	
tlr4bb (Rv)	TGGCGGAGAGAAAAACCAAGA	
dharma (Fw)	CGGAATTCCATCTTAGGAGACACCAGCAGGC	Vector: pBlueScript SK+
dharma (Rv)	CGGGATCCCACGTCGATTCTTGAACCACAC	
frzb (Fw)	CGGAATTCATCTGCGTCCTGGCCTTCGCA	
frzb (Rv)	CGGGATCCTGGGTTTCCCTCAGGGTTACAT	
luciferase (Fw)	CCATCGATTTACCGACGCACATATCGAGGTGGAC	
luciferase (Rv)	CGGAATTCGCAATCCGGTACTGCCACTACTGTTC	
vent (Fw)	CATGAATTCTAGCGGAGAAACTGCACCTG	
vent (Rv)	GTAGGATCCGCATGCAAAATCGCCGGT	
