# Supplementary material for: Determining zebrafish dorsal organizer size by a negative feedback loop between canonical/non-canonical Wnts and Tlr4/NFκB
Source: Nat Commun. 2023 Nov 8;14:7194. doi: 10.1038/s41467-023-42963-3 (PMC10632484; doi:10.1038/s41467-023-42963-3)
Supplement: Supplementary file 3 — Reporting Summary [file 41467_2023_42963_MOESM3_ESM.pdf]

nature portfolio

Corresponding author(s): Tohru Ishitani

Last updated by author(s): Oct 12, 2023

Reporting Summary

Nature Portfolio wishes to improve the reproducibility of the work that we publish. This form provides structure for consistency and transparency in reporting. For further information on Nature Portfolio policies, see our Editorial Policies and the Editorial Policy Checklist.

Statistics

For all statistical analyses, confirm that the following items are present in the figure legend, table legend, main text, or Methods section.

n/a	Confirmed	
☐	☒	The exact sample size (*n*) for each experimental group/condition, given as a discrete number and unit of measurement
☐	☒	A statement on whether measurements were taken from distinct samples or whether the same sample was measured repeatedly
☐	☒	The statistical test(s) used AND whether they are one- or two-sided<br>*Only common tests should be described solely by name; describe more complex techniques in the Methods section.*
☒	☐	A description of all covariates tested
☐	☒	A description of any assumptions or corrections, such as tests of normality and adjustment for multiple comparisons
☐	☒	A full description of the statistical parameters including central tendency (e.g. means) or other basic estimates (e.g. regression coefficient) AND variation (e.g. standard deviation) or associated estimates of uncertainty (e.g. confidence intervals)
☐	☒	For null hypothesis testing, the test statistic (e.g. F, t, r) with confidence intervals, effect sizes, degrees of freedom and P value noted<br>*Give P values as exact values whenever suitable.*
☒	☐	For Bayesian analysis, information on the choice of priors and Markov chain Monte Carlo settings
☒	☐	For hierarchical and complex designs, identification of the appropriate level for tests and full reporting of outcomes
☒	☐	Estimates of effect sizes (e.g. Cohen's *d*, Pearson's *r*), indicating how they were calculated

Our web collection on statistics for biologists contains articles on many of the points above.

Software and code

Policy information about availability of computer code

Data collection	Stereo microscope: MZ16FA (Leica)<br>Confocal laser-scanning microscope: FV3000 (Olympus)<br>qPCR: Mx3000P QPCR system (Agilent)
Data analysis	Imaging data analysis: Fiji/ImageJ<br>Statistical analysis and graphs: Excel 2019 (Microsoft), Prism8 (GraphPad)

For manuscripts utilizing custom algorithms or software that are central to the research but not yet described in published literature, software must be made available to editors and reviewers. We strongly encourage code deposition in a community repository (e.g. GitHub). See the Nature Portfolio guidelines for submitting code & software for further information.

Data

Policy information about availability of data

All manuscripts must include a data availability statement. This statement should provide the following information, where applicable:

- Accession codes, unique identifiers, or web links for publicly available datasets
- A description of any restrictions on data availability
- For clinical datasets or third party data, please ensure that the statement adheres to our policy

All the data supporting this study are available within the article, supplementary information, and source data. Source data are provided with this paper.

Research involving human participants, their data, or biological material

Policy information about studies with human participants or human data. See also policy information about sex, gender (identity/presentation), and sexual orientation and race, ethnicity and racism.

Reporting on sex and gender	not applicable
Reporting on race, ethnicity, or other socially relevant groupings	not applicable
Population characteristics	not applicable
Recruitment	not applicable
Ethics oversight	not applicable

Note that full information on the approval of the study protocol must also be provided in the manuscript.

Field-specific reporting

Please select the one below that is the best fit for your research. If you are not sure, read the appropriate sections before making your selection.

- [x] Life sciences
- [ ] Behavioural & social sciences
- [ ] Ecological, evolutionary & environmental sciences

For a reference copy of the document with all sections, see nature.com/documents/nr-reporting-summary-flat.pdf

Life sciences study design

All studies must disclose on these points even when the disclosure is negative.

Sample size	No statistical methods were used to predetermined sample size. Sample size was chosen based on previous study used the similar methods (Akieda et al. "Cell competition corrects noisy Wnt morphogen gradients to achieve robust patterning in the zebrafish embryo." Nature communications vol. 10,1 4710. 17 Oct. 2019, doi:10.1038/s41467-019-12609-4).
Data exclusions	No data were excluded from the analyses.
Replication	At least two independent experiments were taken to verify the reproducibility of the experimental findings. All experiments were reliably reproduced.
Randomization	Embryos from zebrafish crosses were randomly allocated into experimental groups for injections and chemical inhibitor treatments.
Blinding	The investigators were not blinded to the groups and treatments during experiments,since embryos from zebrafish crosses were genetically uniform and indistinguishable.

Reporting for specific materials, systems and methods

We require information from authors about some types of materials, experimental systems and methods used in many studies. Here, indicate whether each material, system or method listed is relevant to your study. If you are not sure if a list item applies to your research, read the appropriate section before selecting a response.

Materials & experimental systems

n/a	Involved in the study	
☒	☐	Antibodies
☐	☒	Eukaryotic cell lines
☒	☐	Palaeontology and archaeology
☐	☒	Animals and other organisms
☒	☐	Clinical data
☒	☐	Dual use research of concern
☒	☐	Plants

Methods

n/a	Involved in the study	
☒	☐	ChIP-seq
☒	☐	Flow cytometry
☒	☐	MRI-based neuroimaging

Eukaryotic cell lines

Policy information about cell lines and Sex and Gender in Research

Cell line source(s)	HEK293 cell line was gifted from Dr. Kunihiro Matsumoto. Original commercial source was #CRL-1573™, ATCC.
Authentication	To authenticate HEK293 cells, we performed periodic morphology check via microscope. To prevent deterioration of cells, we used the cells at low passage numbers.
Mycoplasma contamination	Cell line tested negative for mycoplasma contamination.
Commonly misidentified lines (See ICLAC register)	No commonly misidentified cell lines were used.

Animals and other research organisms

Policy information about studies involving animals; ARRIVE guidelines recommended for reporting animal research, and Sex and Gender in Research

Laboratory animals	4-12 months old adult zebrafish (Danio rerio) were used to obtain fertilized eggs. Assays were conducted in zebrafish embryos and larvae at 3-27 hpf.<br><br>• Zebrafish wild-type strain (AB), rel mutant,<br>• Tg(6xNFkB-tkP:dGFP),<br>• Tg(OTM:d2EGFP),
Wild animals	We did not use wild animals.
Reporting on sex	Assays were conducted in zebrafish embryos and larvae at 3-27 hpf. At these developmental stages, sex is not yet determined.
Field-collected samples	We did not use field-collected samples.
Ethics oversight	All experimental animal care was performed in accordance with institutional and national guidelines and regulations. The study protocol was approved by the Institutional Animal Care and Use Committee of the respective universities (Osaka University, RIMD Permit# R02-04).

Note that full information on the approval of the study protocol must also be provided in the manuscript.
